# Differential item functioning in the autism behavior checklist in children with autism spectrum disorder based on a machine learning approach

**DOI:** 10.3389/fpsyt.2024.1447080

**Published:** 2024-09-16

**Authors:** Kanglong Peng, Meng Chen, Libing Zhou, Xiaofang Weng

**Affiliations:** ^1^ Rehabilitation Department, Shenzhen Children’s Hospital, Shenzhen, China; ^2^ Rehabilitation Department, Luohu District Maternal and Child Health Care Hospital, Shenzhen, China

**Keywords:** ABC, Rasch model, differential item functioning, ASD, machine learning

## Abstract

**Aim:**

Our study utilized the Rasch analysis to examine the psychometric properties of the Autism Behavior Checklist (ABC) in children with autism spectrum disorder (ASD).

**Methods:**

A total of 3,319 children (44.77 ± 23.52 months) were included. The Rasch model (RM) was utilized to test the reliability and validity of the ABC. The GPCMlasso model was used to test the differential item functioning (DIF).

**Result:**

The response pattern of this sample showed acceptable fitness to the RM. The analysis supported the unidimensionality assumption of the ABC. Disordered category functions and DIF were found in all items in the ABC. The participants responded to the ABC items differently depending not only on autistic traits but also on age groups, gender, and symptom classifications.

**Conclusion:**

The Rasch analysis produces reliable evidence to support that the ABC can precisely depict clinical ASD symptoms. Differences in population characteristics may cause unnecessary assessment bias and lead to overestimated or underestimated symptom severity. Hence, special consideration for population characteristics is needed in making an ASD diagnosis.

## Introduction

Autism spectrum disorder (ASD) is a highly heritable and heterogeneous neurodevelopmental disorder whose symptoms emerge in the early developmental stage and persist along the overall lifespan (1). For now, the specific pathogenesis mechanism underlying ASD is unknown; hence, no comprehensive cure for ASD has been found (2). Timely diagnosis is needed to initiate early interventions, which can lead to more optimal developmental outcomes in individuals with definitive or suspected ASD (3, 4).

A comprehensive ASD diagnosis is established based on the detailed developmental trajectory, clinical observation, and the application of standardized diagnostic instruments (3). As no objective evidence can decide whether autistic traits fulfill the criteria to make an ASD diagnosis or not, the diagnostic decision is mainly built on the clinician’s experience or the patients’ self-perception (1, 5).

To promote diagnostic reliability and validity, clinicians tend to describe autistic symptoms in two dimensions according to the Diagnostic and Statistical Manual of Mental Disorders, 5th Text Revision (DSM-5-TR), including social communication and restricted and repetitive behaviors (6). Studies found that autistic symptoms can be quantitatively rated on three dimensions including social interaction, communication, and restrictive and repetitive behaviors built based on the DSM, 4th Text Revision (DSM-4-TR) (1, 7–9). Findings proposed that the three-dimension rating structure is more optimal compared to others (e.g., DSM-5-TR) (7). Hence, individuals with ASD may present various extreme autistic traits in different dimensions, and clinicians need to decide whether autistic symptoms are merely autistic-like personalities or true autistic symptoms (10). For example, one study found that boys may display more restrictive interest in typical examples presented by clinical assessment (e.g., train, fan, computer, and dinosaurs), but girls may exhibit these interests on more developmentally normative circumscribed interests (e.g., Barbie doll and horse) (11). In fact, autistic symptoms are always heterogeneous, and children with ASD do not necessarily share the same symptomatology or the so-called core symptoms (10). Furthermore, researchers also found that individual autistic profiles built based on DSM-5-TR can be continuously categorized into various subgroups (5). For example, social communication deficits are more common in individuals with ASD who are younger and present lower developmental functioning (12). In contrast, those who are older and with higher developmental functioning tend to present restricted and repetitive behaviors (12). That means the overlap among autistic profiles can be well described by current diagnostic tools, but the variability across different subsamples may jeopardize diagnosis reliability and validity (13). As previous studies report, the symptom diversity may originate from the individual developmental profiles of children with ASD including age, cognition, speech, and language (5, 14). Hence, special considerations are needed in choosing appropriate diagnostic tools to avoid potential bias caused by these latent factors (14).

To address the developmental profile in ASD diagnosis, the International Classification of Disease 11th Revision (ICD-11) tries to describe the autistic traits starting from early childhood, and the ICD-11 defines autistic symptoms as the conflict among limited capacities and social demands (15). The Autism Behavior Checklist (ABC) was built to depict possibly all the main problematic symptoms in individuals with ASD (16). Accumulated evidence indicates that the ABC can be applied in a clinical setting to portray ASD symptomatology with acceptable psychometric properties (17–19). The psychometric research revealed some variability in measurement properties among different subsamples (17, 19, 20). For example, lower sensitivity and specificity were reported in a sample from China (79.31%/70.83%) (20). On the contrary, higher sensitivity (92.11% and 94.7%) and specificity (92.14% and 92.63%) were reported in populations from Egypt and Brazil (17, 19). As known, the ABC was established based on one survey form that contained possibly all autistic behaviors based on clinician experience (21). The ABC tries to depict autistic traits based on five components including relating, sensory, language, and body, object use, and social and self-help (21). To validate the measurement structure, Wadden and Fredrika tried to explore the component structure underlining the ABC, and the results could not confirm the original five-component structure proposed by Krug. Wadden proposed the three dimensions structure, namely, non-responsive, aloof or repetitive, and infantile or aggressive (22). Miranda proposed another five-component structure including non-responsive behavior, infant-like behavior, aggressive behavior, stereotypical behavior, and echolalic speech (23). More carefully designed psychometric studies are needed to investigate the theoretical structure under the ABC to draw a more definitive and clinically useful conclusion (22, 23).

Since timely diagnosis can endow individuals with ASD with appropriate access to early intervention, it is critical to rigorously utilize diagnostic measures to obtain reliable information from individuals with suspected diagnosis of ASD. As we know, Classical Test Theory (CTT) heavily depends on recruiting a sample with typical representative characteristics (24). The psychometric assumption achieved by CTT may vary across different studies due to samples with different characteristics (25). This can be explained by the fact that CTT assumed that measurement accuracy is invariant across all the individuals regardless of personal traits (e.g., gender, age, and ability), and CTT only adopted the total scores to estimate the measured error (26).

Hence, to comprehensively explore the theoretical basis underlying the ABC, this study adopted the Rasch model to elaborate on the item-level psychometric properties in detail. Additionally, this study also tried to describe the magnitude of measurement bias produced by potential variants in clinical applications. We aimed to establish a prediction model for clinicians to identify items that may demand extra consideration to be interpreted or individuals who tend to generate unexpected outcomes in the ABC.

## Materials and methods

### Participants

Participants were recruited from local referral programs of the government service including maternal and childcare service centers, educational institutions, and community agencies. Children with definitive or suspected diagnoses of ASD were referred for comprehensive evaluation through this program. The referred individuals would accept interdisciplinary assessment to achieve a definitive diagnosis of ASD and receive tailored intervention. The comprehensive assessment routinely induces the administration of the ABC and other standardized tools. Prior to administration, all necessary consent forms were obtained from all subjects and/or their legal guardian(s).

### Measure

#### Childhood Autism Rating Scale

The Childhood Autism Rating Scale (CARS) was built to serve as an observation rating scale to depict ASD symptoms through parent/caregiver interviews, observations, and case reviews. The CARS-2 provided an additional version to describe the symptoms of high-functioning individuals with ASD. The original version remained applicable for individuals with ASD aged under 6 years or above 6 years with lower developmental functioning. The CARS consists of 15 items including relation to people, imitation, emotional response, body use, object use, adaption to change, visual response, listening response, sensory, emotional, verbal communication, gesture, activity status, intellectual response, and overall impressions. Each item is assigned a score from 1 to 4 points, where 1 denotes appropriate behavior and 4 denotes behavior severely deviated from normal criteria. The total score is the sum of all items, where higher scores denote more severe ASD symptoms. The CARS was administrated by trained/licensed clinicians or researchers with appropriate training for necessary interviews with parents and caregivers.

One study reported that the CARS can be utilized to categorize the ASD symptom severity into three levels including non-autism, mild-to-moderate autism, and severe autism (27). This tool was utilized to display the overall symptom severity in our sample.

#### Autism Behavior Checklist

The ABC assessment consists of 57 items that involve possibly all typical autistic behaviors. Items are categorized into five components including relating, sensory, language, body use and object manipulation, and social and self-help. Participants were asked according to the item description given by one researcher and rated the item if their children behaved as the item described. Furthermore, each item contained its own score ranging from 1 to 4 points according to the item weights. The weighted score of each item is decided by the occurrence frequency in Krug’s study (21). For example, if item 1 occurred more than item 2, then item 1 is endowed with 4 points, and item 2 is endowed with 2 points. If one item is rated, then the participant gets the according score (e.g., item 1 scores 0/4, and item 2 scores 0/2). The original cut-off score was set at 68, and a total score above 67 indicated severe symptoms or a higher possibility of being diagnosed with ASD (22).

The interrater reliability was 0.85, and the intra-rater reliability was 0.82 (17, 21).

### Data analysis

#### Rasch model

The Rasch model is generally accepted as an augment to Classical Test Theory. The Rasch model converts the raw score summary to its natural logarithm, constructing an interval scale from dichotomous-level observation. Classical Test Theory defines the total score of a set of items as the latent traits of a person, while the Rasch model utilizes the score of items as the Sufficient Statistic for estimating person ability (the latent traits of a person) and item difficulty independently. The Rasch model related the probability of successful (unsuccessful) responses $x_{\upsilon \iota }$ to the difference between person ability $\beta_{\upsilon }\;$ and item difficulty $\delta_{\iota }$ ; it allows us to estimate $\beta_{\upsilon }\;$ and $\delta_{\iota }$ independently from available data, and then we can examine the way these data fit with prediction calculated from the model. The equation is shown below:


$$P\left\{x_{\upsilon \iota }=1|\beta_{\upsilon },\;\delta_{\iota }\right\}=\frac{exp\left(\beta_{\upsilon }-\delta_{\iota }\right)}{\left[1+exp\left(\beta_{\upsilon }-\delta_{\iota }\right)\right]}$$

Hence, the Rasch model is widely used to investigate the properties of each item in scale, and the properties of items include item difficulties, discrimination, and fitness to the hypothetical theoretical model. The Rasch model is widely utilized to test the psychometric properties of commonly used assessment tools including the Test of Infant Motor Development, Motor Proficiency 2nd Edition, and Peabody Developmental Motor Scale (28–32). In this study, the ABC adopted a dichotomous option design (e.g., yes or no). That means if children’s behavior fulfilled the item description, then children score the weighted point, and vice versa. The Rasch model assumed that children with ASD with more severe symptoms may display more problematic behaviors in the ABC. In this study, the weighted point was canceled, and the scoring sheet was rescored by replacing the weighted by 0 and 1 points. Then, the rescored answer sheet contained only dichotomous responses (e.g., 1 and 0).

Hence, our study chose the Rasch model (RM) to examine the construct validity of the ABC.

Data were analyzed using the WINSTEPS software package (http://www.winsteps.com). The Rasch model reveals the relationship between the probability of a specific response and the difference between person ability and item difficulty.

#### Item fitness

The item score and person score were transformed into logit units. Then, the Rasch model was built based on the responses of individuals and the difficulties of items. Item fitness may reflect the prior assumption in the Rasch analysis. All Rasch measurements are established based on the assumption that items should display acceptable fitness to the Rasch model.

Our study adopted the infit mean square (MNSQ) and standardized Z (Zstd) to neutralize the influence of the unexpected response by assigning weight to the calculated residual. The MNSQ describes how much the participants’ response may deviate from the model, and the Zstd denotes how possible the participants may generate unexpected responses. As previous studies suggested, the infit mean square and Zstd should fall within 0.75 to 1.33 and −2 to 2, respectively (33–35). A reasonable differential efficacy was established with a separation index over 2.0, and reliability was supported with an index beyond 0.8 (33–35).

#### Unidimensionality

The residuals calculated based on the difference between the actual and expected performance are used in principal component analysis (PCA). The unidimensional structure is validated if over 40% of the variance of the residual can be explained by the measurement dimension, and the distribution of the residuals that are explained by extra dimension should follow the random characteristics (eigenvalue less than 2.0) (33, 34, 36).

#### Differential item functioning

Further, this study mainly focused on uniform differential item functioning (DIF) to detect the potential variants that may bring bias to the interpretation of the ABC scores. The Rasch model is the most frequently utilized method to identify DIF. However, large sample sizes may limit the statistical power and more easily produce type 1 errors. Furthermore, the Rasch model cannot control the impact of other confounding factors. For example, DIF produced by gender may also be affected by other demographic factors (e.g., age and education). To overcome these limitations, one machine learning method was introduced in our study. This study used a machine learning method to establish the Rasch model with the least absolute shrinkage and selection operator (lasso) penalty to detect the uniform DIF in the ABC. The GPCMlasso R package was utilized to calculate λ, which denotes the influence of the covariance (e.g., age group, gender, and symptom level in this article) on item response probability. Thus, the uniform DIF is confirmed if this lasso coefficient is unequal to zero. In this study, DIF analysis was conducted to test the influence of covariates including gender, age group, and symptom level.

Therefore, the calculation method can be written as follows:


$$\begin{array}{c} log\left(\frac{P\left(Y_{pi}=r\right)}{P\left(Y_{pi}=r-1\right)}\right)\\ =\beta_{i}[\theta_{p}+x_{p}^{T}-\delta_{ir}-(\gamma_{i1}\;*\;Gender+\gamma_{i2}\;*\;AgeGroup\\ +\gamma_{i3}\;*\;Symptomlevel)]. \end{array}$$

In this equation, $\gamma_{in}\left(n=1,\;2,\;3\right)$ denotes the influence of covariates on item i. To modify the original DIF analysis methods (e.g., Welch’s t-test), the GPCMlasso package can test multiple covariates simultaneously and eliminate the potential multicollinearity that may exist among these variables. In this article, the Bayesian information criterion was adopted to screen for the optimal parameter λ.

#### Sample consideration

To obtain 99% confidence that the item calibration (item difficulty measure) is within ±1/2 logit of its robust value and avoid type one errors, a sample between 250 and 500 is recommended (37–39).

## Result

### Demographic data

A sample consisting of 3,319 children and adolescents was involved in this study. **Table 1** presents the demographic data for this sample. The mean age was 44.77 ± 23.52 months, and the gender ratio was 2,645/674 (male/female). Our study tried to recruit a sample with balance in terms of age range designed based on the Chinese Education System (kindergarten, 3–6 years; primary school, 6–12 years; junior high school, 12–15 years; and high school, 15–18 years), but we ultimately obtained a sample of 1,414 children before registration in kindergarten, 1,503 children attending kindergarten, 380 children from primary school, 19 children and adolescents from junior high school, and 3 from high school. In terms of symptom severity, we managed to obtain a sample with a balance in the CARS severity classification as shown in **Table 1**.

**Table 1:** Participant demographic data.

Variables	Mean (SD)/count (%)
Sample	3,319
Gender	
Male	2,645 (79.69%)
Female	674 (20.31%)
Age (months)	
Overall	44.77 (23.52)
0–35/infant	1,414 (42.60%)
36–71/kindergarten	1,503 (45.28%)
72–143/primary	380 (11.45%)
144–180/junior high	19 (0.6%)
179–216/high school	3 (0.07%)
ASD symptoms (CARS classification)	
Symptom level	31.70 (5.37)
Non-autism	1,135 (34.20%)
Mild-to-moderate autism	1,446 (43.57%)
Severe autism	738 (22.23%)

SD, standard deviation; CARS, Childhood Autism Rating Scale; ASD, autism spectrum disorder.

### Person and item mapping and fit statistics

**Figure 1:**
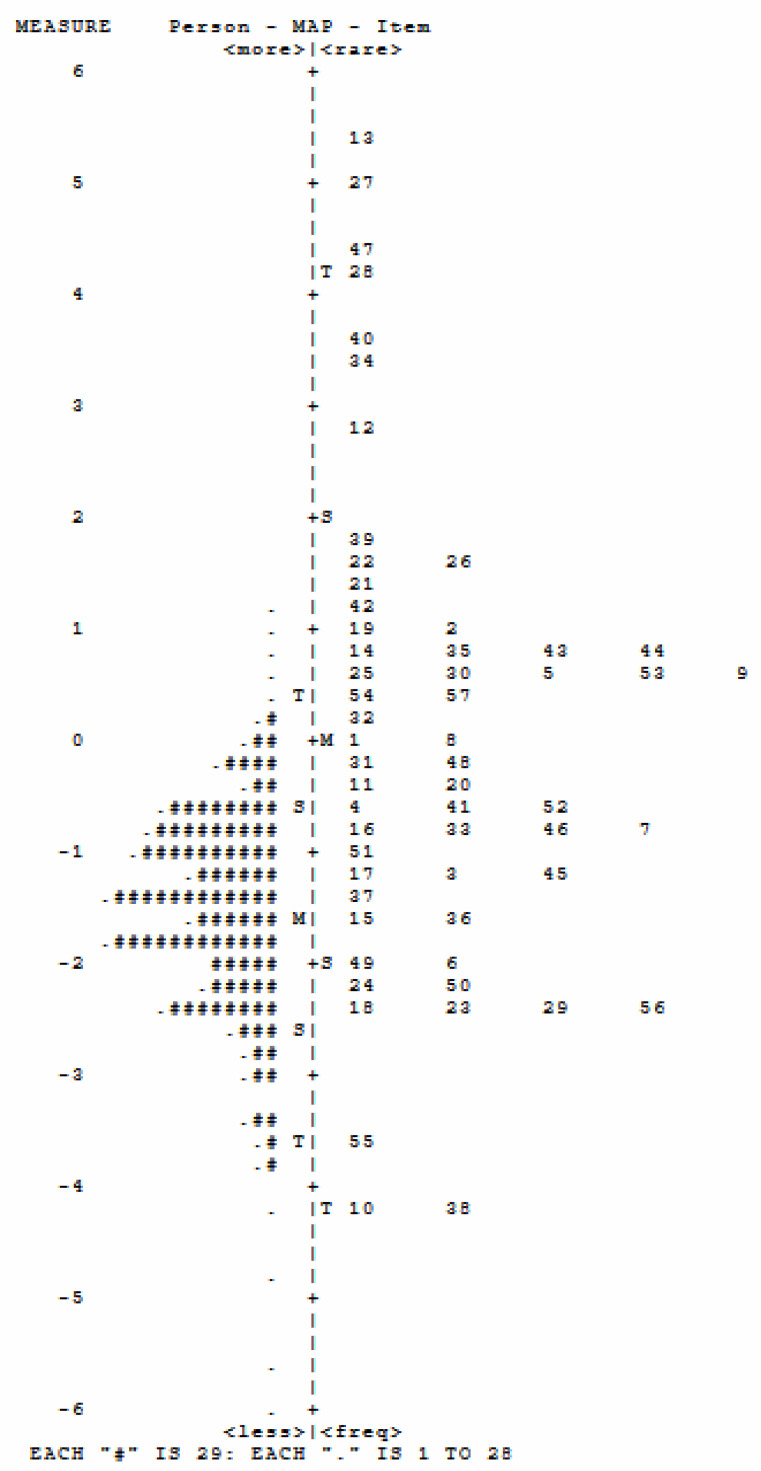
Person–item map of the items in the ABC. ABC, Autism Behavior Checklist. Each "#" represents 29 persons, and each "." represents 1-28 person, for example, "." on the top represents 1-28 children are located above "1" ability level, 2) the number on the right represent the item, for example, ""10 on the botton represents item 10,3) the number on the left side represent the symptom occurence frequency continuum, for exapmle, item 10 on the participants may display this behavior.

**Figure 1** displays the overall view of the item occurrence frequency distribution. In **Figure 1**, the vertical line denotes the frequency continuum, and the upper position stands for less frequent behavior. As shown in **Figure 1**, item 13 (“Does not (or did not as a baby) reach out when reached for.”) was the least frequent behavior. That means an intention to reach for something may be the behavior that is only shown in those with the most severe autistic symptoms. Item 10 (“Seems not to hear (despite normal hearing tests).”) and item 38 (“Has not developed any friendships.”) were the most common behaviors, which means that under-reaction to external stimulation may be the most common symptom in this sample. As the mean item frequency was set at 0 logit, **Figure 1** shows that the majority in this sample may present mild-to-moderate symptoms. Furthermore, **Figure 1** shows that most of the items manage to cover almost the entire scale, and the items are nearly continuously distributed from −2 to 2 logit. That means the ABC can distinguish nearly 76% of the symptom variance (e.g., 2 logit = 12%, and −2 logit = 88% frequency).

The item–person map or the Wright map depicts the item and person distribution along the autistic trait scale constructed based on the ABC. This mapping is built by arbitrarily setting the mean item difficulty to 0. The item–person displays that the ABC items tend to cluster averagely along the 0 logit; hence, individuals with mild-to-moderate symptoms can be depicted more in detail.

The response pattern in this sample shows reasonable fitness to the expectation of the Rasch model (**Table 2**). That means the following analysis results are produced based on solid prior assumptions.

**Table 2:** Fit statistics summary of the ABC.

Fit statistics		Total score	Count	Measure	Infit		Outfit		Real separation	Real reliability
					MNSQ	Zstd	MNSQ	Zstd		
Person	Mean	17	57	−1.56	1	−0.08	1.04	0.09	2.17	0.83
	SD	0.1	0	0.02	0	0.02	0.02	0.02		
Item	Mean	988.5	3,319	0	1	−0.75	1.09	−0.13	18.8	1
	SD	110	0	0.28	0.02	0.7	0.04	0.75		

SD, standard deviation; ABC, Autism Behavior Checklist.

The person reliability and separation index showed that the ABC is efficient enough to distinguish children with ASD with different symptom severity from each other. That means the ABC can capture the inter-person variations in symptom severity rather than other irrelevant behaviors. A value of 0.83 denotes that 83% of the personal variations captured by the ABC are caused by interindividual differences, and 16% is random error. The item reliability and separation index showed that the recruited sample was large enough to figure out the ranking of items on the measurement continuum.

### Evaluation of item fitness

Our analysis detected 29 items that showed misfitting (over or unfitting) to the Rasch model (**Table 3**). That means these items may reflect some behaviors that are not related to symptom severity, or these behaviors tend to happen randomly instead of in a pattern. Among them, only item 8 violated the MNSQ and Zstd criteria [e.g., MNSQ (0.75–1.33) and Zstd (−2 to 2)]. These items may be more suspected as random behaviors rather than autistic traits. In general, the MNSQ denotes the unstandardized residual between the expected and real values, and Zstd presents the standardized residual that tends to cancel the effect of too erratic or robust pattern in this sample. That means the MNSQ indicates the magnitude of the residual, while the Zstd indicates the possibility of the unexpected value. In this case, the residual between expected and real performance was within the normal range, but the response pattern was too robust or erratic. For example, item 3 (“Frequently does not attend to social/environmental cues.”) displays normal MNSQ (e.g., 0.86) but unusual Zstd (e.g., −9.9), which means the reason why children do not respond to external cues may be related to autistic symptom severity, but this behavior can also happen randomly in children with ASD. As another example, item 8 (“Exhibits pronoun reversal (you for I, etc.).”) displays abnormal MNSQ and Zstd simultaneously, and that means an appropriate personal pronoun may not be a suitable behavior to calibrate the symptom severity. This could be explained by the fact that pronoun utilization may be a common problem in children as well.

**Table 3:** Fit statistics for unfitting (overfitting and misfitting) items.

Item	Content	Total score	Total count	Measure	Model S.E.	Infit		Outfit		Corr.
						MNSQ	Zstd	MNSQ	Zstd	
3	Frequently does not attend to social/environmental cues.	1,405	3,319	−1.16	0.04	0.86	−9.9	0.82	−8.34	0.51
4	Does not follow simple commands (sit down, come here, and stand up) given once.	1,022	3,319	−0.58	0.04	0.92	−4.61	0.83	−5.41	0.43
5	Does not use toys appropriately (spins wheels, etc.).	480	3,319	0.51	0.05	0.89	−3.4	0.71	−5.41	0.38
6	Poor use of visual discrimination when learning (fixates on parts of objects such as size, color, and position).	1,957	3,319	−1.96	0.04	0.95	−3.41	0.93	−3.16	0.45
7	Lacks a social smile (may smile out-of-context).	1,181	3,319	−0.83	0.04	0.83	−9.9	0.77	−8.91	0.53
8	Exhibits pronoun reversal (you for I, etc.).	690	3,319	0.02	0.05	1.34	9.9	1.95	9.9	−0.09
10	Insists on keeping certain objects with him/her.	3,009	3,319	−4.22	0.06	0.88	−2.59	0.6	−6.08	0.46
11	Speech is atonal and arrhythmic.	885	3,319	−0.34	0.04	1.25	9.9	1.5	9.9	0.07
15	Does not respond to own name when called out among two or more other names.	1,764	3,319	−1.68	0.04	0.82	−9.9	0.79	−9.9	0.56
16	Lunges and darts about, interrupted by spinning, toe walking, hand flapping, etc.	1,223	3,319	−0.89	0.04	0.89	−7.88	0.84	−6.24	0.47
17	Not responsive to other people's facial expressions or feelings.	1,380	3,319	−1.12	0.04	0.86	−9.9	0.83	−7.83	0.51
18	Seldom uses “yes” or “I”.	2,267	3,319	−2.45	0.04	0.92	−4.13	0.87	−4.93	0.48
19	Having special abilities in one area seems to rule out intellectual disability.	296	3,319	1.1	0.06	1.13	2.65	1.82	8.19	0.01
20	Does not follow simple prepositional commands (e.g., “put the ball in the box”).	887	3,319	−0.35	0.04	0.87	−7.04	0.77	−6.62	0.46
23	Severe temper tantrums and/or frequent minor tantrums.	2,228	3,319	−2.38	0.04	1.16	8.4	1.24	8.66	0.25
29	Gets desired objects by gesturing.	2,192	3,319	−2.32	0.04	0.92	−4.96	0.85	−6.3	0.49
31	Hurts others by biting, hitting, kicking …	831	3,319	−0.25	0.04	1.19	8.81	1.47	9.9	0.11
32	Repeats phrases repeatedly.	575	3,319	0.27	0.05	1.24	7.74	1.78	9.9	0
33	Does not imitate other children at play.	1,156	3,319	−0.79	0.04	0.83	−9.9	0.76	−9.21	0.52
36	Does not wait for needs to be met (wants things immediately).	1,696	3,319	−1.58	0.04	1.05	3.83	1.06	2.86	0.35
37	Cannot point to more than five named objects.	1,521	3,319	−1.33	0.04	0.93	−5.14	0.91	−4.53	0.45
38	Has not developed any friendships.	3,021	3,319	−4.27	0.07	0.89	−2.27	0.61	−5.8	0.45
45	Does not dress self without frequent help.	1,380	3,319	−1.12	0.04	1.14	9.75	1.19	7.75	0.24
48	Echoes questions or statements made by other people.	833	3,319	−0.25	0.04	1.31	9.9	1.6	9.9	0
49	Frequently unaware of surroundings and may be oblivious to dangerous situations.	2,031	3,319	−2.07	0.04	0.86	−9.16	0.81	−9.05	0.53
50	Prefers to manipulate and be occupied with inanimate objects.	2,110	3,319	−2.19	0.04	0.83	−9.9	0.76	−9.9	0.57
52	Frequently has no visual reaction to a “new” person.	1,097	3,319	−0.7	0.04	0.91	−5.67	0.92	−2.69	0.43
53	Gets involved in complicated “rituals” such as lining things up.	445	3,319	0.6	0.05	1.08	2.29	1.32	4.56	0.15
54	Is very destructive (toys and household items are quickly broken)	520	3,319	0.41	0.05	1.08	2.56	1.35	5.46	0.16

Corr, item to total correlation; MNSQ, mean square; Zstd, standardized z.

### Assessment of unidimensionality

The principal component analysis of the residuals revealed that the variance explained by the measure was 60.9%. Three contrasts were detected in the ABC with an eigenvalue over 2. That means the ABC is measuring more than one main principal component (e.g., eigenvalue greater than 2). These extra components indicated those covariates that may jeopardize the measurement accuracy in the ABC. The unexplained ratios of measured variances were 5.3%, 3%, and 2.3%. However, the variance ratio of measures to contrasts was all larger than 3:1 (57/4.92, 57/2.77, and 57/2.13).

To determine which of the ABC items load onto the residual factors, our study arbitrarily set 0.4 as the cutoff value for a meaningful factor loading (**Table 4**) (40, 41).

**Table 4:** Standardized residual loadings for items on contrasts.

Item	Content	Loading	Measure	Infit MNSQ	Outfit MNSQ
1st					
4	Does not follow simple commands (sit down, come here, and stand up) given once.	0.47	−0.58	0.92	0.83
18	Seldom uses “yes” or “I”.	0.58	−2.45	0.92	0.87
20	Does not follow simple prepositional commands (e.g., “put the ball in the box”).	0.51	−0.35	0.87	0.77
29	Gets desired objects by gesturing.	0.74	−2.32	0.92	0.85
37	Cannot point to more than five named objects.	0.73	−1.33	0.93	0.91
56	Uses at least 15 but less than 30 spontaneous phrases daily to communicate.	0.46	−2.38	0.97	0.92
2nd					
3	Frequently does not attend to social/environmental cues.	0.44	−1.16	0.86	0.82
7	Lacks a social smile (may smile out-of-context).	0.46	−0.83	0.83	0.77
24	Actively avoids eye contact.	0.4	−2.15	0.98	0.97
33	Repeats phrases repeatedly.	0.44	−0.79	0.83	0.76
52	Frequently has no visual reaction to a “new” person.	0.42	−0.7	0.91	0.92
3rd					
6	Poor use of visual discrimination when learning (fixates on parts of objects such as size, color, and position).	0.53	−1.96	0.95	0.93
56	Uses at least 15 but less than 30 spontaneous phrases daily to communicate.	0.52	−2.38	0.97	0.92

### Differential item functioning

The DIF analysis was conducted based on the rescored data.

According to Bayesian information criterion (BIC) methods, our results revealed that all items in the ABC display DIF differently in gender, age groups, and symptom classifications (**Table 5**). This implied that these behaviors occur in children and adolescents with ASD with different demographic characteristics differently. Also, these behaviors may be perceived and interpreted differently by the parents and guardians of these children. In the GPCMlasso equation, each group variable is encoded by the corresponding λ, and the predominant variables are set as reference.

**Table 5:** The results of DIF analysis based on lasso coefficients in the GPCMlasso model for variables in the ABC.

Item	Content	Gender	Age group			
			Infant	Kindergarten	Primary	Junior high
Item 1	Whirls self for long periods of time.	0	−0.337	0	0	0
Item 2	Leans a simple task but “forgets” quickly.	0	0	−0.018	−0.318	0
Item 4	Does not follow simple commands (sit down, come here, and stand up) given once.	0	−0.957	0	0.18	0
Item 5	Does not use toys appropriately (spins wheels, etc.).	0	0	0.313	0	0
Item 6	Poor use of visual discrimination when learning (fixates on parts of objects such as size, color, and position).	0	−0.058	0	0.192	0
Item 7	Lacks a social smile (may smile out-of-context).	0	0.045	0	0	0
Item 8	Exhibits pronoun reversal (you for I, etc.).	0	0.968	−0.137	−0.082	0
Item 11	Speech is atonal and arrhythmic.	0	0.926	0	−0.019	0
Item 15	Does not respond to own name when called out among two or more other names.	0	−0.478	0	0.185	0
Item 16	Lunges and darts about, interrupted by spinning, toe walking, hand flapping, etc.	0	0	0.15	0	0
Item 17	Not responsive to other people's facial expressions or feelings.	0	−0.05	0.108	0.059	0
Item 18	Seldom uses “yes” or “I”.	0	−0.867	0	0.107	0
Item 19	Having special abilities in one area seems to rule out intellectual disability.	0	0.929	0	−0.015	0
Item 20	Does not follow simple prepositional commands (e.g., “put the ball in the box”).	0	−0.786	0	0.026	0
Item 24	Actively avoids eye contact.	0	0.374	0	0	0
Item 25	Resists being touched or held.	0.022	−0.199	0	0	0
Item 26	Sometimes, painful stimuli (cuts, injections, and bruises) evoke no reaction.	0	0	0	−0.013	0
Item 29	Gets desired objects by gesturing.	0	−1.321	0	0.175	0
Item 30	Walks on toes	0	−0.089	0.117	0	0
Item 31	Hurts others by biting, hitting, kicking …	−0.087	0	0	0	0
Item 32	Repeats phrases repeatedly.	0	1.066	0	−0.134	−0.034
Item 33	Does not imitate other children at play.	0	0	0.123	0	0
Item 36	Does not wait for needs to be met (wants things immediately).	0	−0.357	0	0.066	0
Item 37	Cannot point to more than five named objects.	0	−1.201	0	0.177	0.032
Item 39	Covers ears at many sounds.	0	0.12	0	−0.252	0
Item 41	Difficulties with toilet training.	0	−0.807	0	0.163	0
Item 42	Uses 5 or fewer words per day spontaneously to communicate wants or needs.	0	−0.291	0	0	0
Item 43	Often frightened or very anxious.	0	0.15	0	0	0
Item 44	Squints, frowns, or covers eyes when in the presence of natural light.	0	0.302	0	0	0
Item 45	Does not dress self without frequent help.	0	0.166	−1.262	0	0
Item 46	Repeats sounds or words repeatedly.	0	0.212	0	0	0
Item 48	Echoes questions or statements made by other people.	0	0	−1.002	−0.613	0
Item 49	Frequently unaware of surroundings and may be oblivious to dangerous situations.	0	0	0.126	0.101	0
Item 53	Gets involved in complicated “rituals” such as lining things up.	0	0	−0.143	0	0
Item 54	Is very destructive (toys and household items are quickly broken).	−0.021	0	0	0	0
Item 55	A developmental delay was identified at or before 30 months of age.	0	−1.15	0	0	0
Item 56	Uses at least 15 but less than 30 spontaneous phrases daily to communicate.	0	−0.687	−0.221	0	0
Item 57	Stares into space for long periods of time.	0	0.142	0	−0.161	0

Non-zero numbers under the group variable represent uniform DIF between subgroups.

DIF, differential item functioning.

To simplify the original formulation, the GPCMlasso model can be written as follows:


$$\begin{array}{c} log\left(\frac{P\left(Y_{pi}=r\right)}{P\left(Y_{pi}=r-1\right)}\right)\\ =[\theta_{p}-(\beta_{i}+\gamma_{i1}\;*\;Gender+\gamma_{i2}\;*\;AgeGroup\\ +\gamma_{i3}\;*\;Symptomlevel)]. \end{array}$$

In this study, the GPCMlasso model set the minorities among the subsamples as dummy code, which means Gender/female, Age group/high school, and Symptom level/severe ASD were equal to 0 in this formulation (e.g., $\gamma_{i1}\;*\;female$ , $\gamma_{i2}\;*\;high\;school$ , and $\gamma_{i3}\;*\;severe\;ASD$ ).

For example, **Table 5** shows that the lasso coefficient for gender was −0.087 in item 31 (“Hurts others by biting, hitting, kicking …”), which means boys are more likely to hurt others physically. For the same $\theta_{p}$ for boys and girls, the item difficulty equals $\beta_{i}-0.087$ for boys and $\beta_{i}$ for girls. The probability equals the difference between $\theta_{p}$ and item difficulty. That means boys have a higher probability of presenting physically harming behaviors compared to girls.

**Figure 2:**
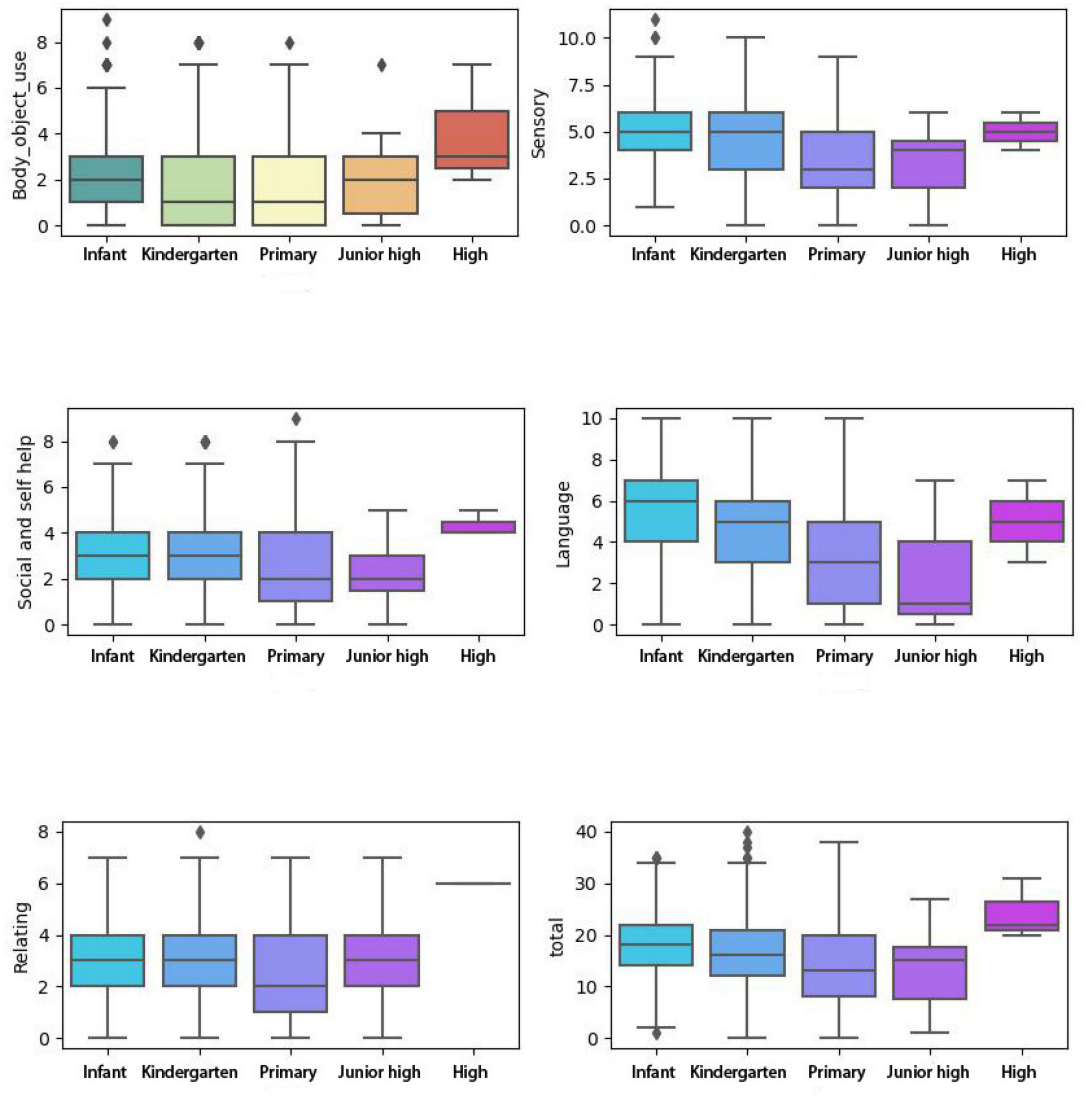
The ABC score comparison in DIF analysis. ABC, Autism Behavior Checklist; DIF, differential item functioning.

This study found no items displaying DIF regarding symptom severity. The ANOVA test was conducted to illustrate the comparison of component scores and total scores among subgroups (**Table 6**, **Figure 2**). **Table 6** shows that overall significant differences among all the subgroups were found. To eliminate the impact brought by the unequal sample sizes among subgroups, the Bonferroni t-test was adopted for the *post-hoc* test.

**Table 6:** The ABC score comparison in DIF analysis.

Components	Gender/mean (SD)		p^2^	Age group					p	(η^2^)^3^	*Post-hoc* comparison test
	Boys	Girls		Infant	Kindergarten	Primary	Junior high	High			
	N^1^ = 2,645	N = 674		N = 1,414	N = 1,503	N = 380	N = 19	N = 3			Bonferroni t-test^4^
Relating	1.88 (1.69)	1.75 (1.63)	0.07	2.02 (1.64)	1.72 (1.69)	1.77 (1.69)	2 (1.83)	4 (2.65)	0.00	0.009	5 > 1, 5<2, 5 > 3, 5 > 4
Sensory	4.59 (1.89)	4.47 (1.87)	0.11	4.71 (1.68)	4.69 (1.92)	3.59 (2.14)	3.42 (1.77)	5 (1)	0.00	0.037	1 > 3, 1 > 4, 2 > 3, 2 > 4
Language	2.7 (1.48)	2.8 (1.48)	0.12	2.69 (1.37)	2.78 (1.53)	2.57 (1.66)	2.16 (1.5)	4.33 (0.58)	0.00	0.004	1 > 2, 1 > 3, 1 > 4, 2 > 3, 2 > 4
Body object use	4.88 (2.26)	5.05 (2.19)	0.08	5.68 (1.79)	4.6 (2.29)	3.44 (2.52)	2.11 (2.11)	5 (2)	0.00	0.116	1 > 2
Social and self-help	2.9 (1.64)	2.99 (1.67)	0.23	2.91 (1.71)	2.97 (1.58)	2.76 (1.65)	2.89 (1.94)	6 (0)	0.00	0.005	–
Total	16.96 (6.77)	17.05 (6.64)	0.74	18.01 (6.03)	16.76 (6.84)	14.14 (7.81)	12.58 (7.06)	24.33 (5.86)	0.00	0.034	1 > 2.1 > 3, 1 > 4, 2 > 3, 2 > 4

^1^N, sample size.

^2^p, p-value.

^3^η^2^, ANOVA η^2^, 0.01 = small effect, 0.06 = medium effect, and 0.14 = large effect.

^4^1 = infant, 2 = kindergarten, 3 = primary, 4 = junior high, and 5 = high.

The result shows that no significant difference was found between boys and girls.

Since we only recruited very few children from junior high school and high school, the *post-hoc* result will be discussed only among infant, kindergarten, and primary children.

We found that children before kindergarten have higher total scores than the other subgroups (e.g., kindergarten and primary). The children before kindergarten presented more obvious behaviors in sensory, language, and body, and object use. No significant results were found in relating and social and self-help.

## Discussion

The aim of this study was to examine the psychometric properties of the ABC by adopting Rasch analysis and machine learning methods. The ABC was developed as a common evaluation tool to be utilized across different populations diagnosed with ASD (e.g., age, gender, and symptom profiles) in clinical settings and research scenarios. The ABC has shown reasonable reliability and validity in previous works. That means the ABC can generate robust results to display the symptom severity in individuals with ASD. In this study, our results try to describe the psychometric properties of the ABC at the item level and address some limitations regarding potential measurement bias. Our findings reveal that the RM is suitable to explain the overall response pattern of individuals with ASD in the ABC evaluation. Furthermore, as a previous study reported, the ABC can explain 60.9% measurement variance, suggesting that the assessment outcomes are calibrated based on a unidimensional construct aiming to depict ASD traits. Our study also revealed some drawbacks regarding the items’ formulation. Differences in group variables may cause potential assessment bias, which can lead to unstable psychometric quality across different subsamples with ASD.

### Measurement properties of the ABC items

The overall response pattern of individuals with ASD shows that children with more severe autistic symptoms displayed more problematic behaviors listed in the ABC. However, two items were not adequately endorsed. Item 13 (“Does not (or did not as a baby) reach out when reached for.”) and item 27 (“Is (or was as a baby) stiff and hard to hold.”) did not get enough response, and only five and seven persons responded to these items. The item-to-total correlation (e.g., −0.01 for item 13 and 0.03 for item 8) suggests that reaching out and body contact may not relate to symptom severity. That is the reason why these two items did not receive enough responses.

### Item difficulty hierarchy and unidimensionality

This study recruited a sample with autistic symptoms ranging from non-autistic to severe autism according to the CARS classification. The item–person map displays that participants mainly represent the population with mild-to-moderate symptoms according to the severity continuum established based on the ABC. That means the ABC can be utilized to quantify the symptom severity in more detail, and the CARS is more suitable for early screening for children with suspected ASD.

For dimensionality analysis, the ABC can explain 60.9% measurement variance. The analysis also reveals three meaningful extra contrasts within the ABC. Hence, we support the multidimensionality assumption as previous studies reported (22, 23).

### Differential item functioning

To date, this study is the first research focusing on the DIF analysis on the ABC. Furthermore, this study also used machine learning methods in the DIF analysis. According to the BIC method, our results reveal that 38 items in the ABC display different DIF in different groups. Our results reveal that the ABC items do not display symptom severity DIF. This implies that parents or caregivers of children with different symptom severities perceive and interpret these items equally. The other results indicate that age groupings and gender can alter the probability of item endorsement in the ABC. The identification of items with DIF emphasizes the need to cautiously interpret the ABC scores at the item level.

For gender DIF, the ABC displays an acceptable ability to capture autistic traits in male and female individuals equally in 54 items (54/57). These behaviors occur equally in children with ASD depending on symptom severity regardless of gender variant. This is in line with previous research that found limited clues for the significant difference in autistic symptoms between male and female individuals with ASD (42, 43).

For age grouping DIF, our finding reveals that the autistic traits captured by the ABC may differ depending on the developmental stage when individuals receive a diagnosis. For example, individuals with more severe symptoms are identified at the age before 3 years or entry into kindergarten in this sample. This is in line with a previous study that found that the Autism-Tics, ADHD, and other Comorbidities inventory (A-TAC) depicts the same performance pattern in individuals with ASD (42).

To date, studies on ASD are commonly recruiting samples that mostly consist of boys; hence, limited research can report the interaction between gender and autistic symptoms (44). Previous findings reveal that autistic symptoms may vary depending on gender diversity (44, 45). For example, item 25 (“Resists being touched or held.”) is more perceived in girls with ASD (e.g., λ^female^ equals 0/λ^male^ equals 0.022). In contrast, item 31 (“Hurts others by biting, hitting, kicking …”) is more commonly seen in boys with ASD (e.g., λ^female^ equals 0/λ^male^ equals −0.087). The explanations for gender DIF may vary across different studies. In this study, we found that boys tend to display more problematic behavior (e.g., hurting others and being destructive), and girls are more rigid in body contact (e.g., being held). These gender diversities have been reported in previous studies as well (43, 46). These studies found that restricted and repetitive behaviors are more perceived in boys with ASD, and girls experience more trouble in sensory sensitivity (43, 46). Previous studies report that female individuals with ASD could display different symptom phenotypes that cannot be comprehensively captured by clinical assessments (42, 46). For example, female individuals with ASD may display symptoms that are more easily accepted (e.g., toys and flowers compared to robots and trains). These interests may lead to misdiagnosis in female individuals with ASD. In some cases, female individuals with ASD cannot receive a timely diagnosis until the autistic traits ultimately cause inevitable problems during adolescence with increasing social demands (47). In this study, we only recruited 675 girls (2,645 boys) with ASD, and this sample may not adequately display the gender diversity in individuals with ASD. Overall, our study found that most symptom topography is shared between boys and girls, and the DIF statistics reflect meaningful variation in three items. The limited evidence indicates that special consideration is needed to interpret the ABC score regarding gender diversity. However, the possible symptom diversity between genders may be adequately captured by the ABC.

Autistic symptoms occur during the whole developmental trajectory, and measuring these symptoms is complex. The overlapping symptom across various spectrums is elaborated by the homogeneity and heterogeneity nature of the autistic traits. The DIF analysis reveals multiple items that demonstrate systematically different measurement properties related to age groups. In line with previous studies, autistic symptoms are more easily identified within 3 years of age (48). In this study, 1,414 (3,319) individuals received ASD diagnosis before 3 years old. Furthermore, 16 items are more easily perceived in individuals within 3 years old compared to other age groups. This can be explained by the salient developmental trajectories within 3 years old, and children with inappropriate growth rates may be more likely to be identified during this period (48). Studies found that atypical developmental curves may be related to ASD diagnosis at the early age (48). In this study, item 55 (“a developmental delay was identified at or before 30 months of age”) was more easily rated in individuals within 3 years old, which is in line with the current assumptions.

Among those inappropriate behaviors, gaze abnormalities, poor response to social stimuli, no social communication, and hypo/hypersensitivity can be early signs of autism. In this study, we found similar evidence. For example, individuals within 3 years old tend to rate in item 6 (“Poor use of visual discrimination when learning (fixates on parts of objects such as size, color, position …”). The behaviors mentioned above are commonly used in ASD measurement and can be noticed in early diagnostic assessments. We also found that these signs are less likely rated as individuals grow. These findings reveal that individuals with ASD are more likely to be noticed early, as they tend to rate more items compared to older subsamples (49). Hence, the timing of receiving the ASD diagnosis may be delayed if individuals get older, leading to a worse prognosis (4, 49).

In this study, we apply the GPCMlasso package as one machine learning method to overcome the multicollinearity problem and covariate calibration that disturbs the previous DIF analysis research. In addition to previous findings, our study reveals that the ABC cannot remain measurement-invariant across gender and age groups.

### Implications for clinical practice

This finding provides preliminary evidence to assess the scale fitness, unidimensionality, and item DIF in the ABC. The results support the conclusion that the ABC is established based on a reasonable measurement structure. However, the results also reveal several shortcomings that may jeopardize the psychometric quality of the ABC. This research reminds the clinician to apply the ABC with rigorous consideration for the potential covariates (e.g., developmental stage) to eliminate possible bias. Notably, a clinical decision is needed when the ABC only provides scores that approach the judgment threshold (e.g., 67 points).

### Study limitation

However, from the statistical perspective, our study fails to involve other possible comorbidities to calibrate the personal variances. Furthermore, the GPCMlasso model is only applicable to uniform DIF; hence, other items with non-uniform DIF cannot be detected. The uniform DIF denotes that the occurrence frequency is not always higher in one subgroup (e.g., infant). For example, one item is more common in infants with lower symptom severity and less likely observed in those with higher symptom severity. The DIF pattern cannot maintain stability along the whole severity continuum (50). Hence, it is more complicated to use the penalized likelihood function to determine items with non-uniform items, even though it may be theoretically feasible.

## Conclusion

Our results support that the ABC is applicable for measuring autistic symptoms in individuals with ASD. Several drawbacks were identified. Items in the ABC display different DIF in corresponding subsamples. Understanding the symptom profile of individuals with ASD in the ABC by focusing on the interaction between the item difficulty and person ability can support more appropriate evaluation and intervention for this population.

## Data Availability

The raw data supporting the conclusions of this article will be made available by the authors, without undue reservation.
